# Beneficial Effects of a Dual Cyclooxygenase Inhibitor−Thromboxane Antagonist in Counteracting High-Fat Diet-Induced Metaflammation

**DOI:** 10.3390/molecules31193403

**Published:** 2026-09-24

**Authors:** Giacomo Einaudi, Elisa Porchietto, Federica Blua, Debora Collotta, Eleonora Aimaretti, Francesca Boccato, Barbara Rolando, Elisabetta Marini, Raffaella Mastrocola, Maria Vittoria Micioni Di Bonaventura, Massimo Collino, Massimo Bertinaria, Gustavo Ferreira Alves, Carlo Cifani

**Affiliations:** 1 Pharmacology Unit, School of Pharmacy, University of Camerino, 62032 Camerino, Italy; giacomo.einaudi@unicam.it (G.E.); mariavittoria.micioni@unicam.it (M.V.M.D.B.); carlo.cifani@unicam.it (C.C.); 2 Department of Neurosciences (Rita Levi Montalcini), University of Turin, 10126 Turin, Italydebora.collotta@unito.it (D.C.); massimo.collino@unito.it (M.C.); gustavo.ferreiraalves@unito.it (G.F.A.); 3 Department of Drug Science and Technology, University of Turin, Via Pietro Giuria 9, 10125 Turin, Italy; federica.blua@unito.it (F.B.); francesca.boccato@unito.it (F.B.); barbara.rolando@unito.it (B.R.); elisabetta.marini@unito.it (E.M.); 4 Department of Clinical and Biological Sciences, University of Turin, 10043 Turin, Italy; eleonora.aimaretti@unito.it (E.A.); raffaella.mastrocola@unito.it (R.M.)

**Keywords:** metaflammation, obesity, cardiometabolic dysfunction, cyclooxygenase-2, thromboxane prostanoid receptor, multi-target drugs

## Abstract

Chronic low-grade inflammation (metaflammation) is a key contributor to obesity-associated metabolic disorders and their cardiovascular complications. This study investigated the effects of CXT29, a novel dual cyclooxygenase-2 (COX-2) inhibitor and thromboxane prostanoid receptor (TP) antagonist, in comparison with its parent compound etodolac, in a murine model of diet-induced metabolic dysfunction. Four-week-old male C57BL/6 mice were fed either a standard diet or a high-fat diet for 18 weeks and subsequently treated with etodolac (20 mg/kg/day) or CXT29 (25 mg/kg/day) by oral gavage for 5 weeks. High-fat-diet-fed mice developed increased body weight, impaired glucose tolerance, altered circulating metabolic hormones, liver dysfunction, steatosis, inflammatory cell infiltration, and systemic inflammation. Both treatments attenuated systemic inflammation and improved the metabolic alterations induced by the obesogenic diet. Notably, CXT29, but not etodolac, attenuated the increase in circulating plasminogen activator inhibitor-1 (PAI-1), a biomarker associated with cardiovascular risk. These findings indicate that pharmacological inhibition of COX-2 improves metabolic dysfunction associated with diet-induced obesity. Furthermore, the additional TP antagonism provided by CXT29 does not compromise the metabolic benefits associate with COX-2 inhibition, while displaying a distinct effect on circulating PAI-1, supporting further investigation of this dual-target approach in obesity-associated metabolic dysfunction.

## 1. Introduction

Metabolic disorders are a wide group of diseases sharing some forms of dysmetabolism leading to conditions such as insulin resistance and obesity, also named diabesity, as well as raised triglycerides, hypercholesterolemia and lowered high-density lipoproteins [1,2]. While some of these conditions are inherited, many of them are caused by hypercaloric diets, sedentary lifestyle and unhealthy habits [3]. The so-called Western diet, named after the eating habits of western countries, is one example of unhealthy diet, comprising high amounts of saturated fats, sugars, processed foods, while having a low quantity of nutrient-dense and fiber-rich foods [4]. Diet-induced metabolic derangements represent a true pandemic, estimated to be affecting up to one quarter of the global population and their prevalence is increasing every year [2,5,6]. In this complicated clinical picture, a key detrimental contributor is the presence of a low-grade chronic inflammatory response, known as metaflammation, that has been speculated to play a central role in the pathogenic mechanism of diabesity [7]. The cyclooxygenase isoenzymes, COX-1 and COX-2, that catalyze the conversion of arachidonic acid to prostaglandins and other lipid mediators, have been demonstrated to be among the most relevant pro-inflammatory pathways contributing to the development of metaflammation and related insulin resistance and lipid accumulation [8]. Prostaglandins are involved in the pathogenesis of dysmetabolism by promoting gluconeogenesis, reducing glucose uptake and inducing insulin resistance [9]. Controversially, some literature data also suggest a positive contribution of COX-produced prostaglandins in mitigating lipid accumulation through browning of the adipose tissue [10,11]. Although an exhaustive understanding of the overall positive or negative contribution of COX in dysmetabolism is still lacking, evidence suggests that inhibition of COX-2, rather than COX-1, plays a pivotal role in preventing diabetes-related complications. This is supported by the high expression of COX-2 in the adipose tissue of obese subjects, and it is known to contribute to both local and systemic inflammation [9,12,13]. The application of nonsteroidal anti-inflammatory drugs (NSAIDs) or selective COX-2 inhibitors (Coxibs), beyond their conventional use, has already been proposed and has given promising results. Interestingly, validation was done in both preclinical and clinical settings where the modulation of COX resulted in favourable outcomes [14]. In clinical trials, COX inhibition in patients suffering from T2DM or metabolic dysfunction-associated steatotic liver disease (MASLD) led to improved insulin sensitivity and better lipid profile [8,15]. In murine models of diet-induced obesity, the use of Coxibs, both as single and combined therapy, led to reduced adiposity and insulin resistance [16,17]. This body of evidence highlights the potential contribution of COX, mainly COX-2, targeting in modulating metaflammation and so leading to favourable metabolic outcomes. However, the potential drug repurposing of Coxibs within the context of metabolic disorders is limited by the cardiovascular risks associated with chronic exposure to this class of drugs and, more widely, to NSAIDs [18,19]. It is in fact known that both NSAIDs and Coxibs administration can lead to increase in thrombotic cardiovascular events [20]. For this reason, recently, new pharmacological approaches aimed to reduce NSAIDs cardiovascular risks have been proposed, based on the development of hybrid drugs. For instance, selective COX-2 inhibitors have been modified with moieties able to release nitric oxide to induce vasodilatation and reduce platelet aggregation [21]. Another approach could be the generation of multi-target drugs able to inhibit COX-2 activity and to exert antagonism at the TP [22,23,24]. This design aims to counteract the imbalance induced by classical inhibitors of COX-2, which limit the production of prostacyclin, a strong vasodilator and the most potent endogenous platelet aggregation inhibitor and consequentially enhance the opposite physiological effects of platelet-derived thromboxane. Moreover, by antagonising TP, the vasoconstrictor activity due to isoprostanes, non-enzymatic products of arachidonic acid oxidation, which act through the TP should be blocked [25,26]. In the past years, we developed dual COX-2 inhibitors and antagonists of the thromboxane prostanoid receptor TP based on the chemical structure of lumiracoxib, a selective coxib approved in 2003 (2006 in Europe), for the treatment of osteoarthritis. However, the obtained compounds were either not potent enough for in vivo studies or not well balanced in the two pharmacological activities. Later, lumiracoxib was withdrawn in most countries owing to hepatotoxic effects, and the FDA finally rejected its approval; therefore, the development of dual COX-2 inhibitors/TP antagonists based on this drug was discontinued. Recently, we modulated the structure of etodolac, an FDA-approved NSAID with preferential COX-2 vs. COX-1 activity [27]. The obtained lead compound, named CXT29, showed favourable pharmacodynamic properties, exerting TP antagonism both in vitro and ex vivo. It also inhibited COX-2 activity in the sub-micromolar range in vitro maintaining the ability to counteract inflammatory pain in vivo. Here, we propose to test the effects of this new dual COX-2 inhibitor and TP antagonist, CXT29, in an in vivo model of diet-induced metabolic derangements. To investigate whether the additional TP antagonism influences the biological responses associated with COX-2 inhibition, the effects observed following CXT29 administration were compared with those evoked by the parent compound etodolac.

## 2. Results

### 2.1. Metabolic Derangements Induced by Dietary Manipulations Were Attenuated by Etodolac and CXT29

Since the oral gavage was selected as the route of administration for CXT29, its stability was first evaluated in simulated gastric fluid, simulated intestinal fluid and human serum at 37 °C. CXT29 was stable during 24 h of incubation in all these media (Supplementary Materials Table S1 and Figures S1–S3). In the in vivo study, BW gain during the 18-week dietary period revealed significant effects of time (*p* < 0.0001) and diet (*p* < 0.0001), as well as a significant time × diet interaction (*p* = 0.0005), indicating that BW trajectories differed between SD and HD mice. Post hoc comparisons showed that HD mice had significantly greater BW gain than SD mice from week 7 through week 18 (week 7, *p* = 0.0251; week 18, *p* = 0.0014). During the 5-week treatment period, BW gain was not significantly affected by time (*p* = 0.7435), whereas a significant effect of treatment was observed (*p* < 0.0001). No significant time × treatment interaction was detected (*p* = 0.9315). Tukey’s multiple-comparisons test showed that at week 23, BW gain was significantly higher in HD mice than in both HD + ETO (*p* = 0.0247) and HD + CXT29 mice (*p* = 0.0342), whereas no significant difference was observed between the two treatment groups (*p* = 0.7687) (Figure 1a). HD mice had elevated fasting blood glucose levels (Figure 1b) compared to SD mice (*p* = 0.0021) and impaired glucose tolerance, as shown by the oral glucose tolerance test (Figure 1c,d) (*p* < 0.0001 AUC of HD vs. SD). HD mice had significantly higher fasting blood glucose levels compared to both etodolac- (*p* = 0.0019) and CXT29-treated (*p* = 0.0236) mice. HD-ETO and HD-CXT29 groups also had improved glucose tolerance compared to HD mice (*p* < 0.0001 vs. HD), without any statistical differences observed between the two treatments (*p* > 0.99 HD-ETO vs. HD-CXT29). Analysis of plasma hormonal levels in HD mice revealed significantly higher concentrations of insulin (*p* = 0.0008 vs. SD) and leptin (*p* = 0.0009 vs. SD) and decreased levels of ghrelin (*p* = 0.0277 vs. SD), glucose-dependent insulinotropic polypeptide (GIP) (*p* = 0.0474 vs. SD) and glucagon-like peptide-1 (GLP-1) (*p* = 0.0464 vs. SD) compared to SD mice, whereas no changes in glucagon concentrations were recorded among all groups (Figure 2a–f). Treatments with etodolac or CXT29 restored hormonal levels to values comparable to those of SD mice.

### 2.2. CXT29 and Etodolac Treatments Reverse HD-Induced Liver Injury and Steatosis

Increased concentrations of alanine transaminase (ALT) (*p* = 0.0104 vs. SD) and aspartate transaminase (AST) (*p* = 0.0082 vs. SD) were detected in the blood of HD mice compared to SD, suggesting hepatic injury. Interestingly, mice treated with CXT29 and etodolac exhibited significantly lower ALT (*p* = 0.0004 for HD-ETO vs. HD, *p* = 0.0013 for HD-CXT29 vs. HD) and AST (*p* < 0.0001 for HD-ETO vs. HD, *p* = 0.0002 for HD-CXT29 vs. HD) levels compared with untreated counterparts, indicating beneficial effects on liver integrity (Figure 3a,b). Oil Red-O staining revealed diffused lipids deposition in the liver of HD mice (Figure 4a), consistent with a significant increase in hepatic triglycerides levels (*p* = 0.0004 vs. SD) (Figure 4b). Notably, triglycerides levels were significantly reduced following the administration of either etodolac or CXT29 (*p* < 0.0001 vs. HD). Histological analysis of liver sections showed normal liver architecture in SD fed mice, while HD mice exhibited focal inflammatory cell infiltrates and diffused lipid droplets accumulation. These features were absent in the livers of HD mice receiving either treatment (Figure 4c). As shown in Figure 4d, the enzymatic activity of MPO, a marker of neutrophils infiltration, was significantly increased in HD-fed mice compared with the SD group (*p* = 0.0008) and was drastically reduced after 5 weeks of treatment with either etodolac or CXT29 (*p* < 0.0001 vs. HD). Plasma levels of tumor necrosis factor-α (TNF-α) (*p* = 0.0468 vs. SD, interleukin-6 (IL-6) (*p* = 0.008 vs. SD) and interleukin-17 (IL-17) (*p* = 0.0032 vs. SD) (Figure 5a–c) were elevated in HD-fed mice compared to SD and significantly decreased following pharmacological manipulations (*p* < 0.05 for HD vs. HD + ETO and HD + CXT29).

### 2.3. Effects of CXT29 and Etodolac on Circulating Resistin and PAI-1

Lastly, we measured the systemic concentrations of resistin and plasminogen-activator inhibitor-1 (PAI-1), markers associated with cardiovascular risk [28,29]. As shown in Figure 6, HD feeding induced a significant increase in both resistin (*p* < 0.0001 vs. SD) and PAI-1 (*p* < 0.0001 vs. SD). Both pharmacological treatments were able to reduce plasma resistin levels (*p* = 0.0006 for HD-ETO vs. HD, *p* = 0.0001 for HD-CXT29 vs. HD), with no significant difference between etodolac and CXT29 (*p* = 0.2339). In contrast, the diet-induced increase in PAI-1 was attenuated by CXT29 administration (*p* < 0.0001 vs. HD), whereas etodolac did not significantly affect PAI-1 concentrations (*p* = 0.3624 vs. HD). Interestingly, the levels of PAI-1 were significant lower in HD-CXT29 compared to HD-ETO (*p* = 0.0033).

## 3. Discussion

In recent years, evidence has demonstrated that chronic consumption of reducing sugars and saturated fatty acids activates inflammatory pathways that contribute to the development of metabolic dysfunction [30,31].

Thus, pharmacological approaches aimed at counteracting metaflammation have been explored in preclinical settings and anti-inflammatory drugs already approved for chronic inflammatory diseases have been considered interesting for potential “drug repurposing” [32]. However, the well-known cardiovascular toxicity of both Coxibs and NSAIDs may dramatically exacerbate the cardiovascular risk intrinsically associated with the diet-induced metabolic derangements, thus limiting their therapeutic potential in this context. In fact, Coxibs do not affect platelet function, since platelets do not express COX-2, while NSAIDs, except for low-dose aspirin, fail to confer cardiovascular protection because of their transient and incomplete inhibition of platelet COX-1-dependent TXA2 [33]. In fact, a low concentration of TXA2 can induce a complete platelet activation in the presence of sub-threshold concentrations of other platelet agonists. Furthermore, experimental evidence suggests that nonplatelet TXA2 generation represents an independent risk factor for long-term cardiovascular mortality [34,35]. In this context, TP antagonists may offer a promising alternative by inhibiting the effects of both platelet- and nonplatelet-derived TXA2 without impacting on vascular prostacyclin.

Here, we demonstrated that mice consuming an HD diet and treated with etodolac or CXT29 exhibited a reduction in BW, an improvement in glucose tolerance and showed a restored metabolic hormonal profile, without any significant difference between the two treatments. While most of the experimental evidence in animal models of diet-induced metabolic disorders has highlighted the role of pharmacological or genetic COX inhibition on adipose tissue inflammation, our study primarily investigated the liver condition [12,36,37]. The liver plays a critical role in lipid metabolism, synthesis, storing and breakdown for energy, and the export of fats and cholesterol via lipoproteins and bile. Prolonged excessive fat intake leads to hepatic fat accumulation, insulin resistance and progressive liver dysfunction [38]. Consequently, immune cells infiltrate the liver, which evokes inflammation and exacerbates hepatic damage, creating a vicious cycle that can lead to the development of MASLD in patients [39]. In line with literature data showing that COX-2 inhibition ameliorates hepatic inflammation, here we observed marked improvements in liver pathology after treatment [9,40]. Given the established COX-inhibitory activity of etodolac and CXT29, the observed improvements in hepatic inflammation and metabolic parameters may partly reflect modulation of COX-dependent pathways. It should be noted, however, that etodolac is generally regarded as a preferential, rather than highly selective, COX-2 inhibitor; accordingly, the contribution of COX-2 inhibition specifically cannot be inferred from the effects of etodolac in the present experimental setting. Moreover, COX activity and downstream prostanoid production were not directly assessed, and therefore a causal contribution of COX inhibition to the observed effects cannot be established from the present data.

Reduction of inflammation is critical for improving overall function: in our experimental settings, reduced circulating transaminases and hepatic lipid accumulation were observed in treated mice. Moreover, consistent with data showing that COX-2 inhibition, via suppression of prostaglandins production, can ameliorate the IRS-1 pathway both in vitro and in vivo, we observed an improvement in glucose homeostasis [8,41]. Thus, our findings are consistent with previous evidence indicating that modulation of COX-dependent pathways may influence diet-induced metabolic dysfunction. The largely comparable effects observed with etodolac and CXT29 indicate that the structural modification introduced in CXT29 did not compromise its overall pharmacological efficacy in this experimental setting.

Our results are in line with previous observation that terutroban, a selective TP antagonist, ameliorates renal damage in uninephrectomised obese Zucker rats without affecting BW, hyperglycemia and dyslipidemia [42].

However, when we extended the exploration to circulating biomarkers associated with cardiovascular risk, we observed differences between CXT29 and etodolac. Both etodolac and CXT29 reduced resistin levels, a marker associated with cardiovascular disease events, with no significant difference between the two treatments [43]. In contrast, CXT29, but not etodolac, attenuated the HD-induced increase in circulating PAI-1 levels, while the levels in HD-ETO mice were significantly higher compared to those in CXT29-treated mice. PAI-1 is known to inhibit plasmin production and thus promotes the accumulation of fibrin clots. Elevated circulating PAI-1 has been associated with an increased risk of cardiovascular events [28]. In particular, the use of PAI-1-directed monoclonal antibody has been proved to promote thrombolysis and to limit thrombus extension in experimental models of canine thrombosis. Targeting PAI-1 in different animal models of cardiovascular disease has provided substantial evidence of beneficial effects in vivo, with several inhibitors moving forward to clinical trials [44,45]. The selective reduction in circulating PAI-1 levels recorded in CXT29-treated mice may be related to its TP antagonism activity, since this effect was not detectable following etodolac administration. This possibility is consistent with previous evidence linking TP receptor activation to PAI-1 regulation in vascular and renal cells. Indeed, TP activation has previously been shown to increase PAI-1 expression in glomerular mesangial cells, an effect prevented by a TP antagonist [46]. Moreover, pharmacological TP receptor antagonism has been reported to exert protective effects in experimental models of diabetes and atherosclerosis, supporting a broader role for TP signaling in vascular and metabolic dysfunction [47,48,49]. Genetic TP deficiency was also shown to attenuate renal dysfunction in a mouse model of diabetic nephropathy [50]. The diabetic condition was also accompanied by increased COX-2 expression and TXA2 levels in the renal cortex, further supporting a link between enhanced prostanoid production and TP-dependent pathological responses [50].

TXA2/TP signaling has been implicated in the regulation of prothrombotic and inflammatory processes, providing a potential mechanistic basis for the differential effect of CXT29 on PAI-1. Nevertheless, our findings do not establish a causal relationship between TP antagonism and PAI-1 reduction, since TXA2 production, TP receptor signaling and tissue-specific regulation of PAI-1 were not directly assessed. Future studies should therefore determine whether CXT29 directly modulates PAI-1 expression through TP-dependent signaling and identify the cellular sources contributing to the changes in circulating PAI-1. Furthermore, the difference between CXT29 and etodolac was limited to a single cardiovascular risk-related biomarker.

The combination of COX inhibition and TP receptor antagonism in CXT29 also raises the possibility that these pharmacological activities may interact. COX enzymes regulate the biosynthesis of multiple prostanoids, including TXA2, whereas TP receptor antagonism can prevent cellular responses to TXA2 and other TP agonists. Thus, TP antagonism may further limit TP-dependent pro-inflammatory signaling that persists despite COX inhibition. Future mechanistic studies addressing prostanoid production, COX activity and TP receptor signaling will be required to clarify whether COX inhibition and TP antagonism exert interconnected or largely independent effects.

Overall, our findings show that the dual COX inhibitor/TP receptor antagonist CXT29 retains the metabolic, hepatic and anti-inflammatory effects observed with etodolac in HD-fed mice. Across most of the assessed outcomes, no significant differences were detected between the two treatments, whereas a differential effect was observed only for circulating PAI-1 levels. This differential effect suggests that the pharmacological profiles of CXT29 and etodolac may not completely overlap. However, given the broad range of endpoints assessed, this and other individual biomarker findings should be interpreted with appropriate caution and warrant confirmation in specifically designed studies.

## 4. Materials and Methods

### 4.1. Materials

Unless otherwise stated, all reagents were purchased from the Merck Group (Darmstadt, Germany).

### 4.2. CXT29 Synthesis

The desired methyl 2-(8-benzyl-1-ethyl-6-fluoro-1,3,4,9-tetrahydropyrano[3,4-b]indol-1-yl)acetate (CXT29) was synthesised according to the synthetic route depicted in Scheme 1. Since a multigram amount of CXT29 was required for in vivo studies, a modification of the originally employed route was implemented [27]. The commercially available 2-bromo-4-fluoroaniline was submitted to the classical diazotisation/reduction procedure employing sodium nitrite and tin (II) chloride in HCl 37% to afford the desired hydrazine that was isolated as the corresponding hydrochloride **2**. This intermediate was used as the starting material for a Fisher-type cyclisation using 2,3-dihydrofuran as the electrophile to afford 2-(7-bromo-5-fluoro-1H-indol-3-yl)ethan-1-ol (**3**). The chromatographic purification of derivative **3** was particularly complex, allowing isolation of a 60% yield of pure indole together with some fractions containing some impurities. An overall yield of 23.4% was obtained over two steps. After purification, compound **3** was submitted to an Oxa-Pictet Spengler cyclisation with methyl 3-oxopentanoate (**4**) using boron trifluoride diethyl etherate as the activating agent to afford methyl 2-(1,8-dibromo-6-fluoro-1,3,4,9-tetrahydropyrano[3,4-b]indol-1-yl)acetate (**5**). The intermediate **5** was heated with bis(pinacolato)diboron using catalytic tris(dibenzylideneacetone)dipalladium(0), tricyclohexylphosphine as the ligand and potassium acetate to afford the boronate **6** in good yield. This derivative is stable enough to be purified by flash chromatography and can be stored at −18 °C for a few days. The boronate **6** was then used as the substrate for a Suzuki reaction with benzyl bromide (**7**). The reaction was conducted in THF/water using (1,1′-bis(diphenylphosphino)ferrocene)palladium(II) dichloride as the catalyst and Na_2_CO_3_ as the base to afford the desired product **8** in 80% yield. Finally, the desired compound CXT29 was obtained by treatment of **8** with 3M LiOH solution.

### 4.3. Animals and Experimental Procedures

The in vivo experimental procedures were approved by the Animal Welfare Body (Organismo Preposto al Benessere Animale, OPBA) of the University of Turin, Turin, Italy and by the Italian Ministry of Health (approval n. 855/2021-PR) in keeping with the European Directive 2010/63/EU on the protection of animals used for scientific purposes, as well as the Guide for the Care and Use of Laboratory Animals.

This study was conducted using four-week-old C57BL/6j (Charles River Laboratories, Calco, Italy) males with a basal weight of 15–20 g, maintained in conventional housing conditions, at environmental temperature 25 ± 2 °C and a light/dark cycle 12/12 h automatically controlled. Animals were assigned a unique identification number from 1 to 40 according to the order in which they were selected from the housing cages. The animals were co-housed for one week prior to the start of the experiment and assigned to either a standard purified diet SD (ssniff^®^, Soest, Germany, E15051-04 EF R/M Control, 5% fat) (*n* = 10), or a high-fat diet HD (ssniff^®^, Soest, Germany, E15772-34 EF D12331 mod. Surwit 35.7% fat) (*n* = 30). Animals were randomly allocated to the SD or HD dietary groups using a random-number generator. Following random allocation, baseline BW was assessed and, when necessary, group assignments were adjusted to ensure comparable mean BW between the SD and HD groups. After 18 weeks of dietary intervention, mice on the HD were randomly assigned, following the same procedure, to three treatment groups (*n* = 10 per group). Group allocations were subsequently adjusted, when necessary, to ensure comparable mean BW across the three groups: HD mice receiving vehicle (oral gavage), HD + Eto mice treated with etodolac (20 mg/kg daily, by oral gavage), and HD + CXT29 mice treated with an equivalent dose of CXT29 (25 mg/kg daily, by oral gavage), all for the subsequent five weeks. During the 5-week treatment period, SD mice received the same vehicle by daily oral gavage as the HD vehicle group. Group allocation was performed prior to initiation of the pharmacological treatments, with BW taken into consideration to ensure comparable mean BW among the three HD groups. Predefined exclusion criteria included premature death, humane endpoint criteria or failure of the experimental procedures. No animals met any exclusion criteria, and all animals completed the experimental protocol. OGTT, molecular and histological analyses were performed by investigators blinded to group allocation. Sample size was determined during experimental planning using G*Power software, within the framework of the experimental protocol approved by the Italian Ministry of Health (Approval No. 855/2021-PR). Details of the power analysis are provided in Section 4.10.

Mice stayed on their respective dietary regiments for a total of 23 weeks; this protocol was based on previous animal studies showing that similar compositions and kinetics of dietary manipulation resulted in robust BW gain and induction of insulin resistance [51,52,53]. Etodolac was administered orally at 20 mg/kg/day, a dose selected based on preclinical studies demonstrating its efficacy in murine models [54,55]. As no previous in vivo dose-ranging data were available for CXT29, its dose was determined on an equimolar basis relative to etodolac. Considering the higher molecular weight of CXT29 (367.4 g/mol) compared with etodolac (287.4 g/mol), 25 mg/kg/day of CXT29 was calculated to be approximately equimolar to 20 mg/kg/day of etodolac. This dose was therefore used for the comparative in vivo study. Both etodolac and CXT29 were dissolved in a solution of Methocel A15LV 3% in PBS pH 7.4, which was also used as vehicle to treat mice in the HD group. BW was recorded weekly.

### 4.4. Oral Glucose Tolerance Test (OGTT)

One day before the end of the experiment, an OGTT was performed in all mice after an overnight fasting period. A 30% glucose solution was administered at the concentration of 2 g/kg by oral gavage. Blood was obtained from the tail vein once before the glucose administration and after 15, 30, 60 and 120 min. The glucose concentration was measured with a conventional glucometer (GlucoMen LX kit, Menarini Diagnostics, Grassina, Italy).

### 4.5. Blood and Organ Collection

On the last day of the experiment, mice were anaesthetised using isoflurane and euthanised by cardiac exsanguination. Blood samples were collected in micro-tubes with EDTA (0.75 mg EDTA/mL blood) and centrifuged to obtain plasma. Liver and skeletal muscle samples were harvested in micro-tubes both with and without optimal cutting temperature compound and stored at −80 °C until analysis.

### 4.6. Hormone and Cytokine Analysis

Plasma insulin, leptin, ghrelin, glucagon, GIP, GLP-1, resistin and PAI-1 levels were measured using a Luminex suspension bead-based multiplexed Bio-Plex 3D system (Bio-Rad Laboratories, Hercules, CA, USA) via the Bio-Plex Pro Mouse Diabetes 8-Plex Assay (Bio-Rad Laboratories 171F7001M) following the manufacturer’s instructions.

Similarly, the TNF-α, IL-6 and IL-17 plasma levels were measured via the Bio-Plex Pro™ Mouse Cytokine Th17 Panel A 6-Plex (Bio-Rad Laboratories #M6000007NY).

### 4.7. Biochemical Analysis

Liver damage markers were assayed with kits (FAR-diagnostics, Pescantina, Italy) measuring AST (#7036) and ALT (#7018) concentrations in the plasma. Hepatic concentrations of triglycerides (#7137) were measured using reagent kits according to standard enzymatic procedures.

### 4.8. Histological Examination of Liver Sections with Haematoxylin and Eosin and Oil Red Staining

Frozen liver sections conserved in optimal cutting temperature compound were cut in a cryostat (7 μm in thickness) and fixed in acetone for 2 min at −20 °C. Prior to staining, tissue sections were assessed for preservation quality based on the integrity of tissue architecture and the absence of major freezing damage, tissue disruption, folds or cracks that could interfere with histological evaluation and colourant deposition. Sample assessment and selection were performed blinded to group allocation. Based on these criteria, the number of adequately preserved samples varied, with two groups having as few as five. Therefore, a subset of five samples per group were selected for subsequent analysis to ensure a balanced sample size across experimental groups. No samples were excluded based on histological findings or treatment-related characteristics.

To observe the accumulation of inflammatory infiltrates with haematoxylin and eosin, liver sections were stained for 5 min with Mayer’s haematoxylin solution and with eosin solution (0,5% *v*/*v*) for 1 min. Sections were then brought to pure alcoholic solution, fixed in xylene solution for 2 min and slides were mounted using coverslips with DPX mounting medium. To assay neutral lipid accumulation with Oil Red staining, sections were fixed in 4% formaldehyde for 2 min and saturated with Oil Red-O solution (0.3% *w*/*v* in 60% isopropyl alcohol solution) for 10 min. Sections were subsequently counterstained with Mayer’s haematoxylin solution for 5 min and they were mounted using aqueous glycerol mounting medium. For both assays, sections were evaluated in a blinded manner; stained tissues were viewed under an Olympus Bx4I microscope (10 and 40× magnification) with an AxioCamMR5 photographic attachment (Zeiss, Göttingen, Germany). For each animal, 10 sections were examined, with multiple microscopic fields assessed per section.

### 4.9. Myeloperoxidase (MPO) Activity Assay

The MPO activity assay was performed as previously described [56]. Briefly, about 100 mg of liver samples was homogenised in 500 μL (1:5 *w*/*v*) of 20 mM PBS (pH 7.4) on ice and then centrifuged. The supernatants were discarded and the pellets were resuspended in 500 μL of HTAB solution (0.5% HTAB in 50 mM PBS, pH 6.0) on ice. After a second centrifugation, the supernatants were collected and used to measure the MPO activity; 30 μL was added with 180 μL of H_2_O_2_ (0.08 M H_2_O_2_ in PBS, pH 5.4) and 20 μL of 3,3′,5,5′-tetramethylbenzidine (final concentration 1.6 mM). The assay measures the H_2_O_2_-dependent oxidation of 3,3′,5,5′-tetramethylbenzidine that, in its oxidised form, has a blue colour, detected spectrophotometrically. A 96-well plate was incubated for 10 min at 37 °C and read at 650 nm. The final supernatant was also used to quantify the protein content using a Bicinchoninic Acid assay (Pierce Biotechnology Inc., Rockford, IL, USA) following the manufacturer’s instructions. MPO activity was expressed as optical density (O.D.) at 650 nm per mg of protein.

### 4.10. Statistical Analysis

Sample size was calculated using G*Power 3.1 software [57], using an F-test (ANOVA: fixed effects, omnibus, one-way), with an α level of 0.05 and 80% power. The calculation was based on experimental data previously published by our research group in a comparable mouse model of high-fat-diet-induced metabolic dysfunction, using circulating GIP levels as a representative metabolic parameter [51]. An effect size (f) of 0.631 was obtained, resulting in a sample size of 10 animals per group.

Longitudinal data [BW and OGTT] are expressed as mean ± SEM of *n* = 10 mice per group. Results were analysed using a mixed-effects model, with time, group and their interaction as fixed effects, followed by Bonferroni-adjusted multiple comparisons when significant. Other data are presented as box-and-whisker plots, where the central line represents the median, the box represents the interquartile range (Q1-Q3) and the whiskers indicate the minimum and maximum values, independently of the statistical test applied. The data distribution was verified by a Shapiro−Wilk normality test and the homogeneity of variances by a Bartlett test. The statistical analysis was performed by one-way ANOVA for parametric data; when a significant overall effect was detected, this test was followed by Bonferroni’s post hoc test. For data that were found not to be normally distributed (Glucagon, GIP, IL-17, TNF-α), non-parametric statistical analysis was applied through a Kruskal−Wallis test followed by Dunn’s post hoc test when a significant overall effect was detected. A *p*-value < 0.05 was considered to be significant. Statistical analysis was performed using GraphPadPrism^®^ software version 7.05 (San Diego, CA, USA).

## 5. Conclusions

We here extended previous findings supporting the role of pharmacological COX-dependent pathways in counteracting metaflammation, leading to improved hepatic function, enhanced glucose homeostasis and reduced hepatic triglycerides. The dual COX/TP inhibitory profile of CXT29 produced metabolic, hepatic and anti-inflammatory effects broadly comparable to those observed with etodolac in HD-fed mice, suggesting that the structural modification conferring TP receptor-antagonist activity to CXT29 did not compromise the beneficial pharmacological profile of its parent compound. CXT29, but not etodolac, attenuated the HD-induced increase in circulating PAI-1. This isolated finding, based on a single risk-related biomarker, suggests that the two compounds may not have completely overlapping pharmacological profiles and provides a rationale for their more comprehensive characterisation. Since our study was limited to circulating biomarkers and did not include functional vascular, thrombotic or atherosclerotic assessments, future studies incorporating such evaluations will be needed to determine whether the reduction in PAI-1 is associated with functional cardiovascular effects. Furthermore, food and energy intake were not assessed in the present study; therefore, the contribution of altered energy intake to the observed reduction in BW gain cannot be excluded. Direct assessments of COX activity, prostanoid biosynthesis and TP receptor signaling will also be required to identify and mechanistically interpret any additional differences between CXT29 and etodolac. Such studies will help define the pharmacological positioning of dual COX-2/TP receptor antagonism and establish whether this approach may offer clinically relevant advantages over conventional COX inhibitors in obesity-associated metabolic dysfunction.

## Figures and Tables

**Figure 1:**
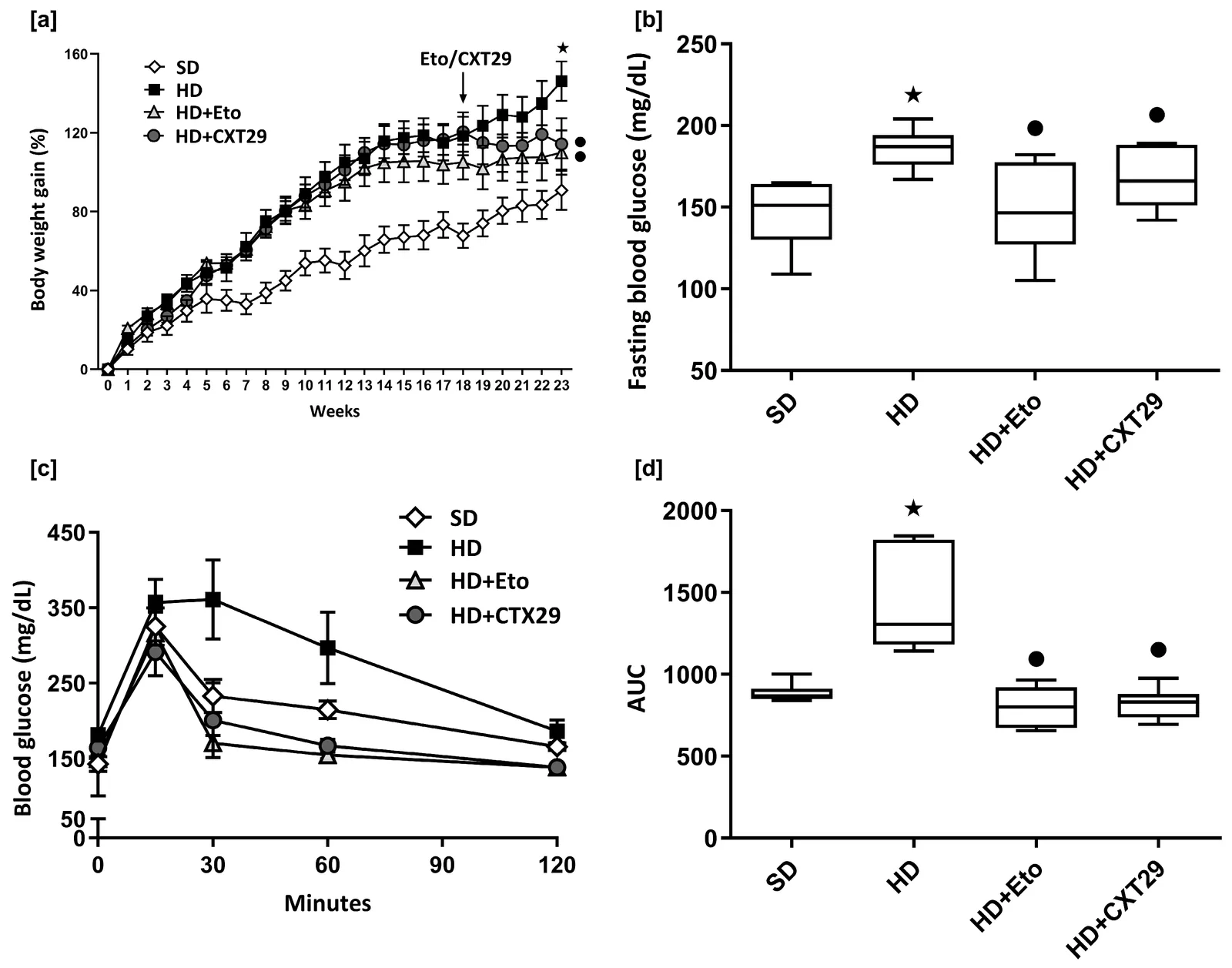
Effects of diet and etodolac (Eto) or CXT29 treatment on body weight (BW) and glucose tolerance at 23 weeks. Mice were randomly assigned to either standard (SD) or high-fat-diet (HD) regimens and starting from week 18, they were treated daily for 5 weeks with vehicle, etodolac (20 mg/kg p.o.) or CXT29 (25 mg/kg p.o.). BW was measured weekly for 23 weeks and is reported as percentage of weight gain from week 0 (**a**). Fasting blood glucose (**b**) was measured at week 23. The oral glucose tolerance test (OGTT) was performed in overnight-fasted mice, and glucose levels are presented as plotted values over time (**c**) and as Area Under the Curve (AUC) (**d**). Data (**a**,**c**) are expressed as mean ± SEM. Data in (**b**,**d**) are presented as box-and-whisker plots, where the central line represents the median, the box represents the interquartile range (Q1–Q3) and the whiskers indicate the minimum and maximum values. Longitudinal data (**a**,**c**) were analysed using a mixed-effects model (REML), with time, group and their interaction as fixed effects, followed by Bonferroni-adjusted multiple comparisons when significant. Fasting blood glucose (**b**) and OGTT AUC (**d**) were analysed by one-way ANOVA followed by Bonferroni’s multiple-comparisons test when significant. ★ *p* < 0.05 vs. SD and • *p* < 0.05 vs. HD.

**Figure 2:**
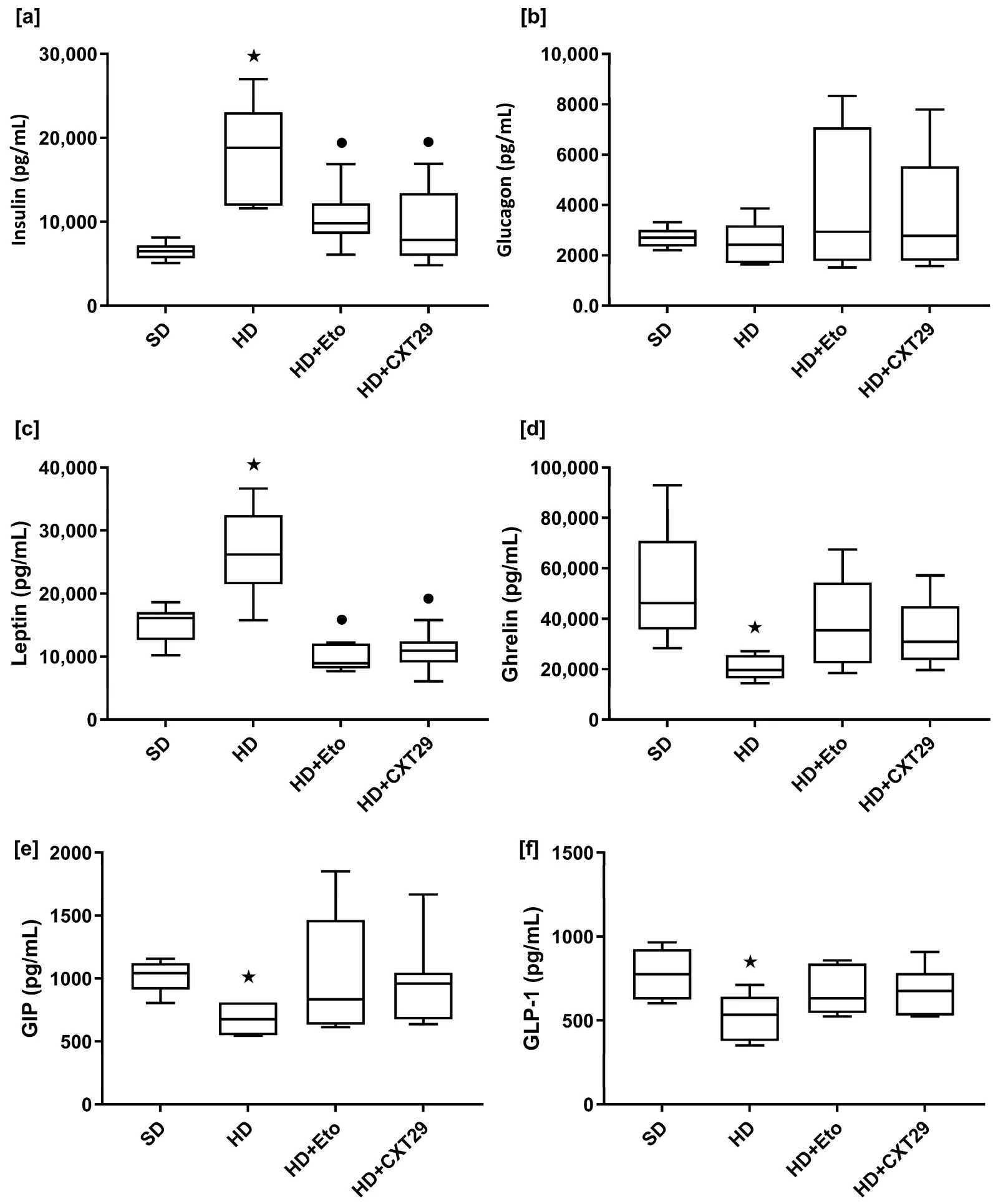
Effects of diet and etodolac (Eto) or CXT29 treatment on plasma concentrations of hormones. Mice were randomly assigned to either standard (SD) or high-fat-diet (HD) regimens and starting from week 18, they were treated daily for 5 weeks with vehicle, etodolac (20 mg/kg p.o.) or CXT29 (25 mg/kg p.o.). After 23 weeks of dietary intervention and respective treatments, plasma samples were collected from mice to measure the concentrations of insulin (**a**), glucagon (**b**), leptin (**c**), ghrelin (**d**), GIP (**e**), and GLP-1 (**f**). Data are presented as box-and-whisker plots (*n* = 10 mice per group), where the central line represents the median, the box represents the interquartile range (Q1–Q3) and the whiskers indicate the minimum and maximum values. Statistical analysis was performed by one-way ANOVA followed by Bonferroni’s post hoc test (**a**,**c**,**d**,**f**) or Kruskal−Wallis test followed by Dunn’s post hoc test (**b**,**e**) for non-parametric data. ★ *p* < 0.05 vs. SD and • *p* < 0.05 vs. HD.

**Figure 3:**
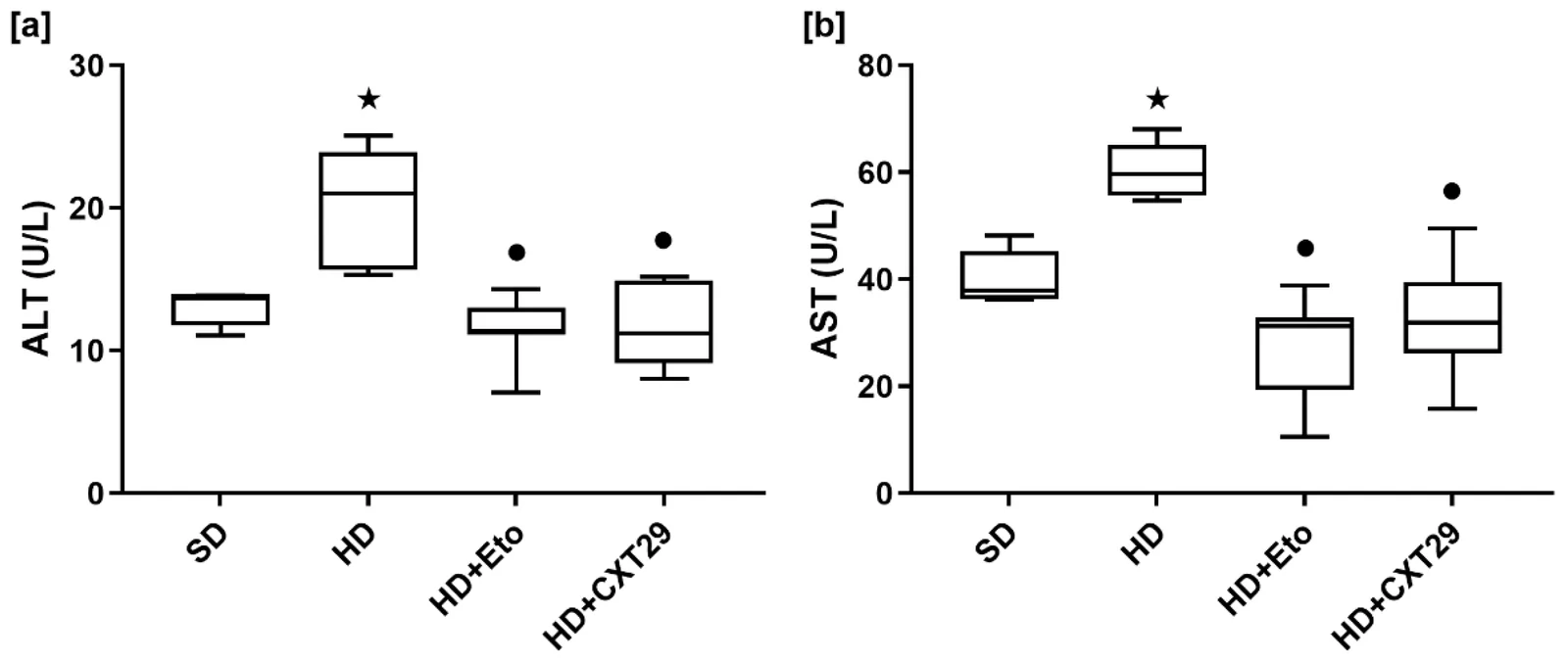
Effects of diet and etodolac (Eto) or CXT29 treatment on plasma concentrations of transaminases. Mice were randomly assigned to either standard (SD) or high-fat-diet (HD) regimens and starting from week 18, they were treated daily for 5 weeks with vehicle, etodolac (20 mg/kg p.o.) or CXT29 (25 mg/kg p.o.). Plasma concentrations of ALT (**a**) and AST (**b**) were measured. Data are presented as box-and-whisker plots (*n* = 10 mice per group), where the central line represents the median, the box represents the interquartile range (Q1–Q3) and the whiskers indicate the minimum and maximum values. Statistical analysis was performed by one-way ANOVA followed by Bonferroni’s post hoc test. ★ *p* < 0.05 vs. SD and • *p* < 0.05 vs. HD.

**Figure 4:**
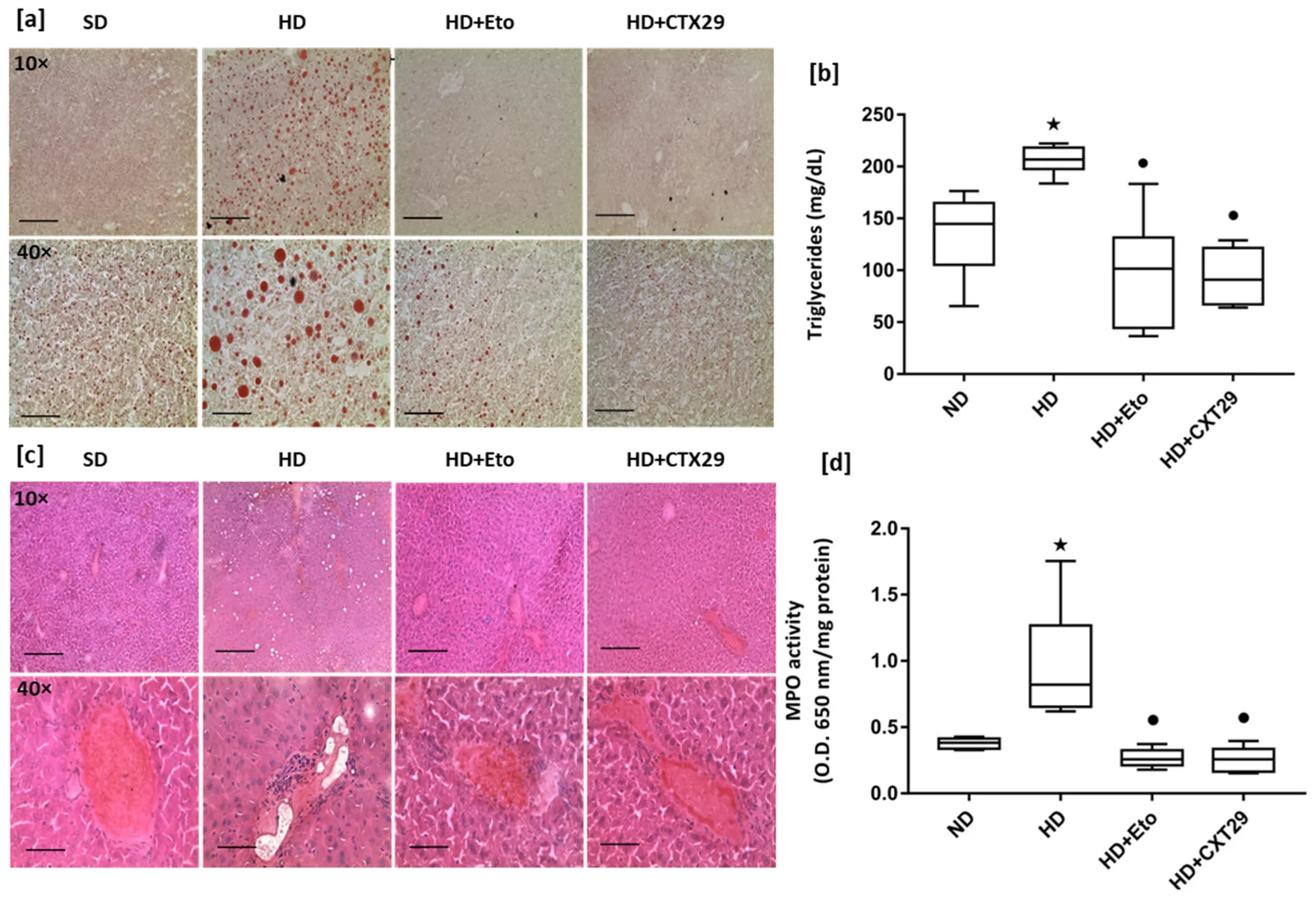
Effects of diet and etodolac (Eto) or CXT29 treatment on the liver. Mice were randomly assigned to either standard (SD) or high-fat-diet (HD) regimens and starting from week 18, they were treated daily for 5 weeks with vehicle, etodolac (20 mg/kg p.o.) or CXT29 (25 mg/kg p.o.). Mice livers were collected and analysed for neutral lipids accumulation by Oil Red staining. Representative photomicrographs from 5 animals per group were acquired at 10× (scale bar = 100 μm) and 40× (scale bar = 25 μm) magnification (**a**). For triglycerides content, the concentrations were determined using a colorimetric kit (**b**). Liver sections were also cut and assayed with hematoxylin and eosin staining (**c**). Myeloperoxidase (MPO) activity was measured through a colorimetric assay (**d**). Data are presented as box-and-whisker plots (*n* = 10 mice per group), where the central line represents the median, the box represents the interquartile range (Q1–Q3), and the whiskers indicate the minimum and maximum values. Statistical analysis was performed by one-way ANOVA followed by Bonferroni’s post hoc test. ★ *p* < 0.05 vs. SD and • *p* < 0.05 vs. HD.

**Figure 5:**
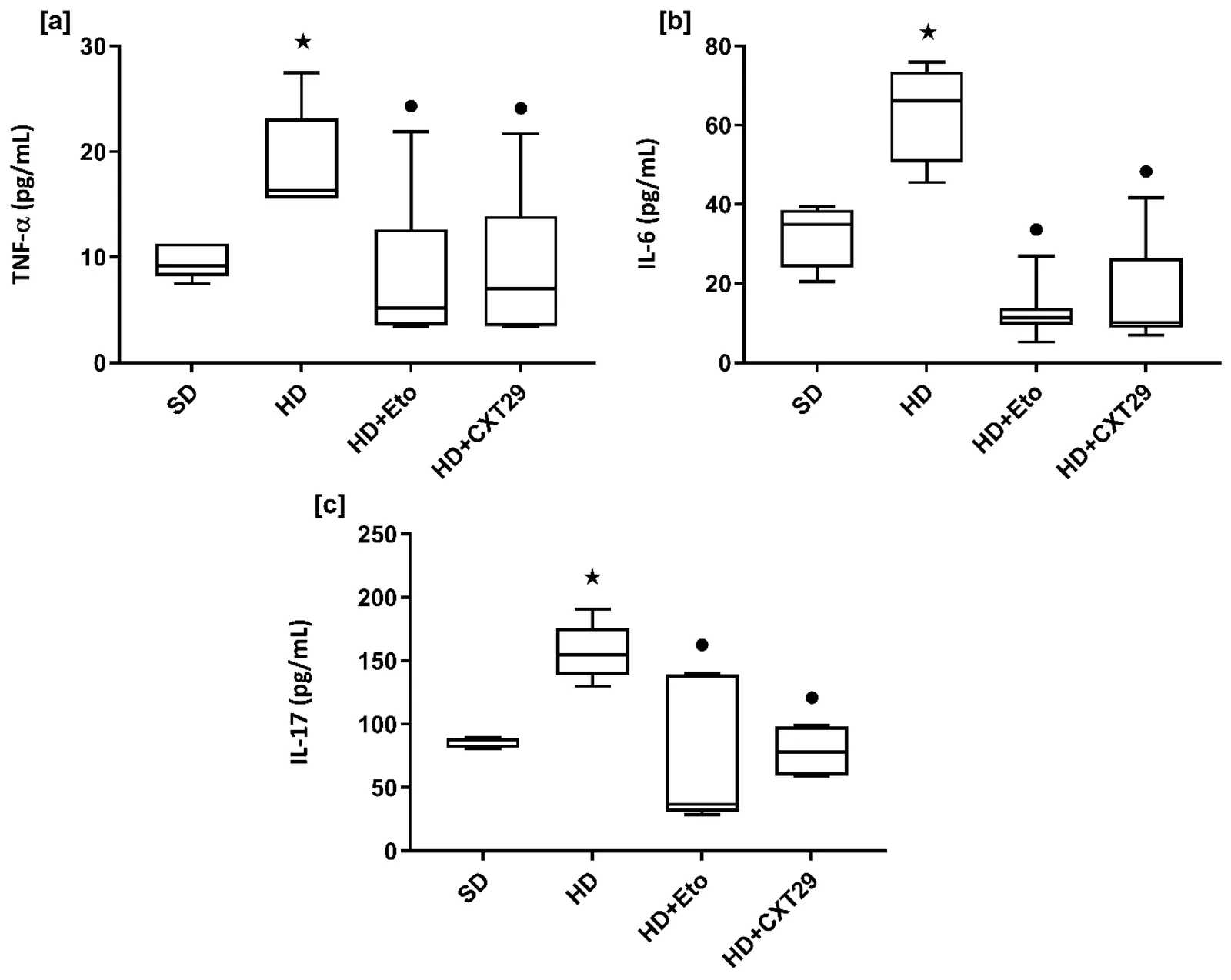
Effects of diet and etodolac (Eto) or CXT29 treatment on plasma concentration of inflammatory cytokines. Mice were randomly assigned to either standard (SD) or high-fat-diet (HD) regimens and starting from week 18, they were treated daily for 5 weeks with vehicle, etodolac (20 mg/kg p.o.) or CXT29 (25 mg/kg p.o.). Mice blood was collected and plasma concentrations of TNF-α (**a**), IL-6 (**b**), and IL-17 (**c**) were determined. Data are presented as box-and-whisker plots (*n* = 10 mice per group), where the central line represents the median, the box represents the interquartile range (Q1–Q3) and the whiskers indicate the minimum and maximum values. Statistical analysis was performed by one-way ANOVA followed by Bonferroni’s post hoc test (**b**), or Kruskal−Wallis test followed by Dunn’s post hoc test (**a**,**c**) for non-parametric data. ★ *p* < 0.05 vs. SD and • *p* < 0.05 vs. HD.

**Figure 6:**
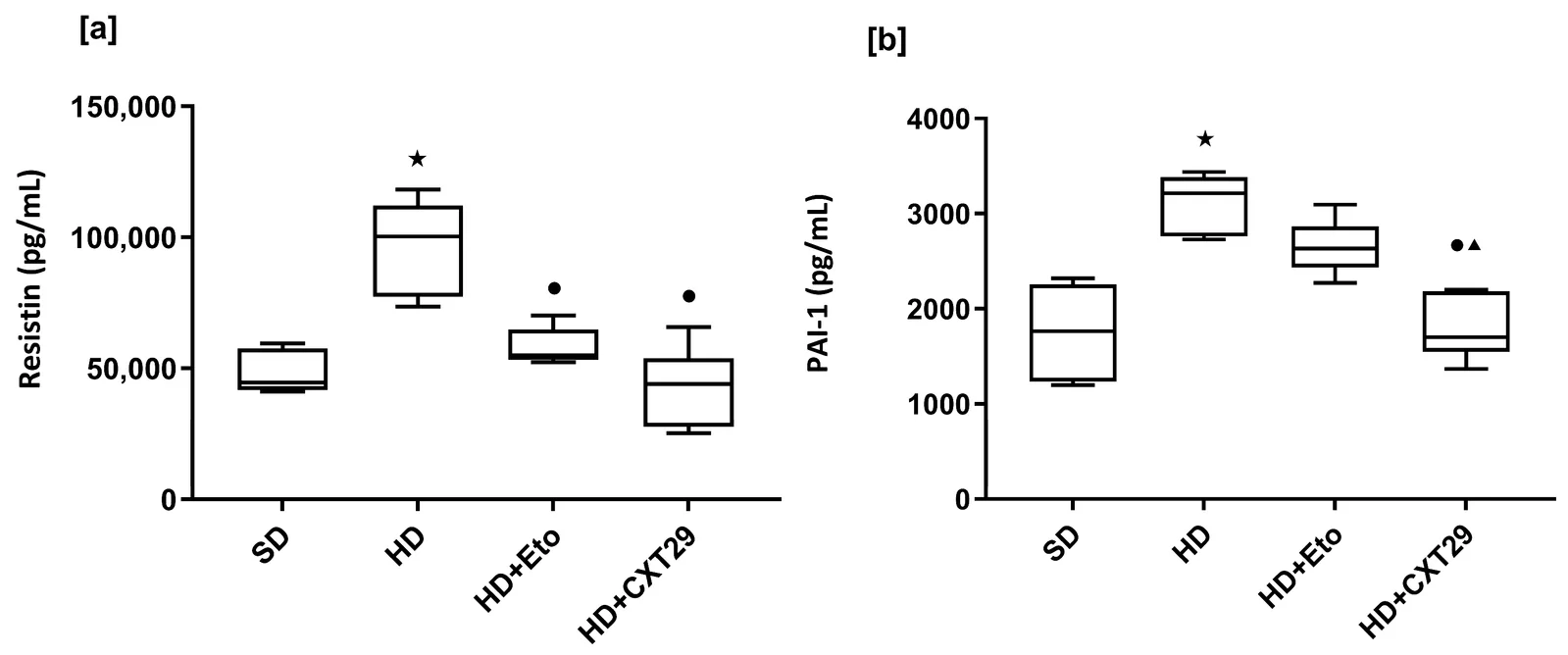
Effects of diet and etodolac (Eto) or CXT29 treatment on plasma concentrations of circulating biomarkers associated with cardiovascular risk. Mice were randomly assigned to either standard (SD) or high-fat-diet (HD) regimens and starting from week 18, they were treated daily for 5 weeks with vehicle, etodolac (20 mg/kg p.o.) or CXT29 (25 mg/kg p.o.). Plasma concentrations of resistin (**a**) and PAI-1 (**b**) were determined. Data are presented as box-and-whisker plots (*n* = 10 mice per group), where the central line represents the median, the box represents the interquartile range (Q1–Q3) and the whiskers indicate the minimum and maximum values. Statistical analysis was performed by one-way ANOVA followed by Bonferroni’s post hoc test. ★ *p* < 0.05 vs. SD and • *p* < 0.05 vs. HD ▲ *p* < 0.05 vs. HD + Eto.

**Scheme 1:**
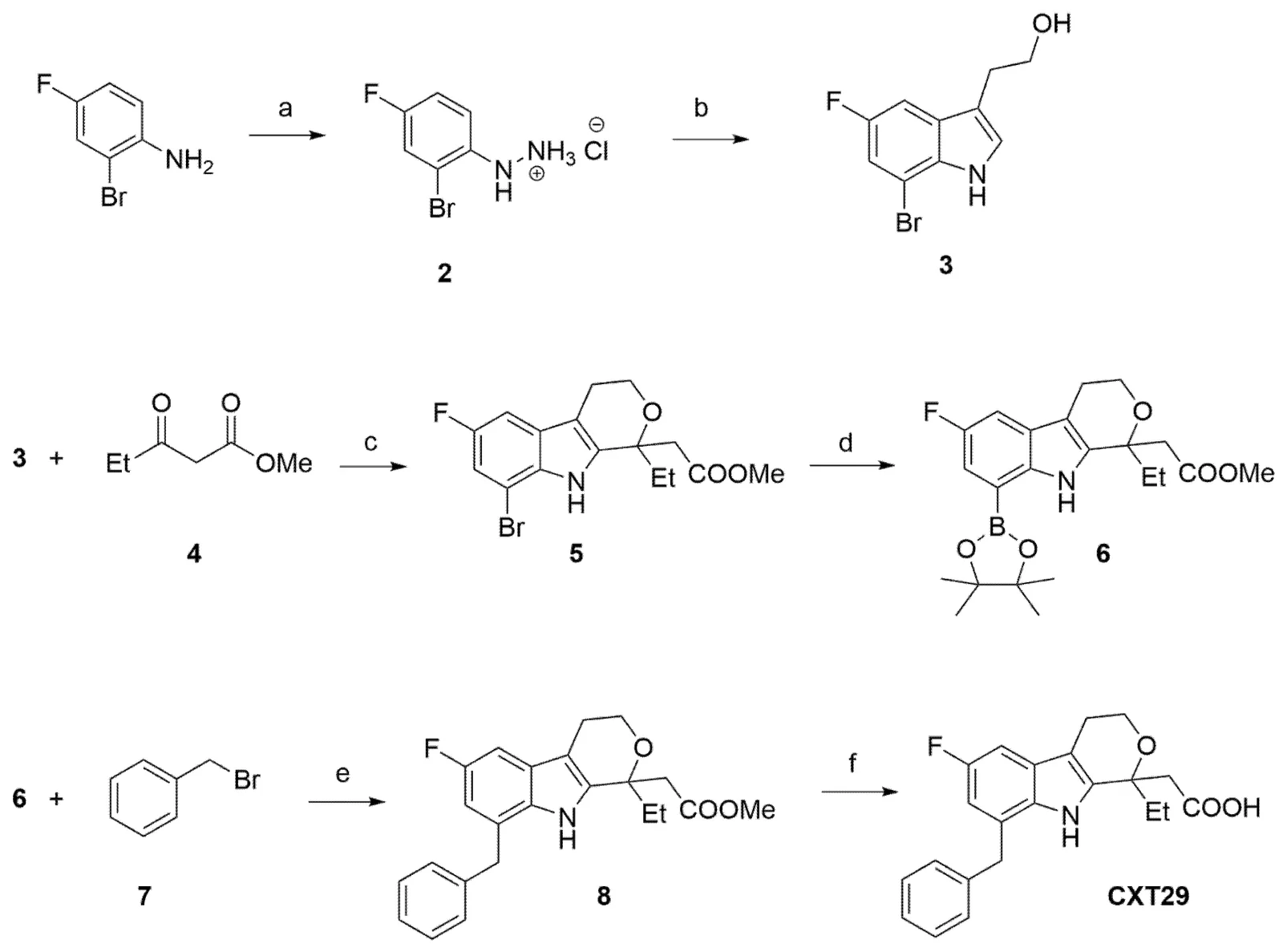
Synthesis of CXT29. Reagents and conditions: (a) (i) NaNO_2_, HCl 37%, 0 °C, 30 min; (ii) SnCl_2_·2H_2_O, HCl 37%, 0 °C, 1 h-3 h; (b) 2,3-dihydrofuran, 1,4-dioxane/H_2_O 10:1, 95 °C, 4 h, (39% over two steps); (c) BF_3_·Et_2_O, dry DCM, rt, 5 h, (60%); (d) bis(pinacolato)diboron, Pd_2_(dba)_3_, PCy_3_, CH_3_COOK, 1,4-dioxane, 100 °C, 16 h (81%); (e) benzylbromide, Pd(dppf)Cl_2_, Na_2_CO_3_, THF/H_2_O 1/1, 40 °C, 16 h (80%); (f) LiOH 3M, 1,4-dioxane, rt, 16 h, (46%).

## Data Availability

The original contributions presented in this study are included in the article/Supplementary Material. Further inquiries can be directed to the corresponding author.

## Supplementary Materials

The following supporting information can be downloaded at: https://www.mdpi.com/article/10.3390/molecules31193403/s1, Figure S1: Stability of CXT29 in SGF; Figure S2: Stability of CXT29 in SIF; Figure S3: Stability of CXT29 in human serum. Table S1: Stability of CXT29 in SGF, SIF and human serum. Section S1: Synthetic procedures.

## Abbreviations

ALT	Alanine transaminase
AST	Aspartate transaminase
BW	Body weight
COX	Cyclooxygenase
Coxib	Selective COX-2 inhibitor
GIP	Glucose-dependent insulinotropic polypeptide
GLP-1	Glucagon-like peptide-1
HD	High-fat diet
IL-17	Interleukin-17
IL-6	Interleukin-6
IRS-1	Insulin receptor substrate-1
MPO	Myeloperoxidase
MASLD	Metabolic dysfunction-associated steatotic liver disease
NSAIDs	Nonsteroidal anti-inflammatory drugs
OGTT	Oral glucose tolerance test
PAI-1	Plasminogen-activator inhibitor-1
SD	Standard diet
TNF-α	Tumor necrosis factor-α
TP	Thromboxane prostanoid receptor
TXA2	Thromboxane A2
