# Supplementary material for: Beneficial Effects of a Dual Cyclooxygenase Inhibitor−Thromboxane Antagonist in Counteracting High-Fat Diet-Induced Metaflammation
Source: Molecules. 2026 Sep 24;31(19):3403. doi: 10.3390/molecules31193403 (PMC13665871; doi:10.3390/molecules31193403)

# Supplementary materials

## Section S1. Synthetic procedures

### Material and methods

Thin-layer chromatography (TLC) was run on 5 x 20 cm plates with 0.25 mm layer thickness (Fluka) and visualised by UV inspection ($\lambda_1$ = 254 nm, $\lambda_2$ = 366 nm) and/or by detection using chemical reagents as $KMnO_4$ (0.5 g in 100 mL of NaOH 0.1 N) and 2,4 dinitrophenylhydrazine (6 g in 60 mL of $H_2SO_4$, 40 mL of $H_2O$ and 200 mL of EtOH). Anhydrous sodium sulfate ($Na_2SO_4$) was used as drying agent for the organic phases.

Dry dichloromethane ($CH_2Cl_2$) was obtained by refluxing over $P_2O_5$ under $N_2$, distilled and stored with molecular sieves (4 Å). Dry tetrahydrofuran (THF) and dry diethyl ether were obtained by refluxing over Na (indicator: benzophenone) under $N_2$. Flash chromatography purifications were performed using *Fluka* silica gel with 60 mesh particles. Mixtures were concentrated under reduced pressure (*in vacuo*) using rotary evaporator apparatus. ESI spectra were recorded on a *Micromass Quattro API micro (Waters Corporation, Milford, MA, USA), mass spectrometer.* $^{1}$H and $^{13}$C NMR spectra were registered on *Jeol ECZ 600 M30* at 600 MHz or 151 Mhz. Chemical shifts (δ) are given in ppm, calibrated to solvent signal as internal standard, and the coupling constants (J) in Hertz (Hz). The following abbreviations are used: s, singlet; d, doublet; t, triplet; q, quartet; dd, double doublet; m, multiplet. Semi-preparative HPLC purifications were carried out on a Varian Pro-Star 210 chromatograph equipped with a variable wavelength detector (Prostar 325). The chromatography was performed using a 5 µM particle size Hibar LiChrosper C18 endcapped prepacked column (250 x 25 mm), with a flow rate of 20 mL/min; UV detection was performed at 226 and 240 nm. Samples of the mixture were dissolved (ca 0.5%) in mobile phase. The following abbreviations are used: petroleum ether (PE); ethyl acetate (EtOAc); methanol (MeOH); tetrahydrofuran (THF); dimethyl sulfoxide (DMSO); trifluoroacetic acid (TFA), polyethylene glycol (PEG), tricyclohexylphosphine ($PCy_3$); thin layer chromatography (TLC); nuclear magnetic resonance (NMR); mass spectrometry (MS); High performance liquid chromatography (HPLC), room temperature (RT).

The purity of target compounds was assessed by RP-HPLC on a HP1100 chromatograph system (Agilent Technologies, Palo Alto, CA, USA) equipped with a quaternary pump (G1311A), a membrane degasser (G1379A), a diode-array detector (DAD) (G1315B) integrated in the HP1100 system. Data analysis were processed by HP ChemStation system (Agilent Technologies). The analytical column was a LiChrosper 100 C18-e (250 x 4.6 mm, 5 µm) (Merck KGaA, 64271 Darmstadt, Germany) eluted with $CH_3CN/H_2O$ + 0.1% TFA in the ratio appropriate for each compound. All compounds were dissolved in the mobile phase at a concentration of about 0.1 mg/mL and injected through a 20 µL loop. HPLC retention times ($t_R$) were obtained at flow rates of 1.0 mL/min; the column effluent was monitored at 226, 240 and 280 nm referenced against 800 nm. The purity of compounds was evaluated as a percentage ratio between the areas of the main peak and of possible impurities at the three wavelengths and also using DAD purity analysis of the chromatographic peak. The HPLC purity of all the intermediates (**3, 5, 6, 8**) was found to be greater than 97%; the purity of the final compound (CXT29) was greater than 99%.

### 2-(7-bromo-5-fluoro-1H-indol-3-yl) etan-1-ol (3).

4-fluoro-2-bromoaniline (8.00 g, 44.4 mmol) was added to HCl 37% (27 mL) and kept under mechanical stirring at 0 °C for 30 min. To the obtained suspension an aqueous solution of $NaNO_2$

(6.13 g, 88.9 mmol) was added dropwise. When the addition was over, the reaction mixture was left under stirring at 0 °C for further 30 min. The mixture was added to a suspension containing $SnCl_2 \cdot 2H_2O$ (25.22 g, 133 mmol) in HCl 37% (13.3 mL) and left under mechanical stirring for 2h at 0 °C. The mixture was filtered and the obtained solid was treated with 4 M NaOH and extracted with $CH_2Cl_2$ (5 x 70 mL). The organic phase was dried ($Na_2SO_4$) and the hydrazine was converted into the corresponding hydrochloride by addition of 6 M HCl in 1,4-dioxane. The solvent was removed under reduced pressure and the obtained solid was dried overnight in a desiccator over $P_2O_5$ to afford 8.51 g of the desired hydrazine hydrochloride **2** which was used for the next step without further purification.

The hydrazine hydrochloride **2** (8.51 g, 35.2 mmol) was dissolved in 1,4-dioxane containing 10% of water (50 mL) and 2,3-dihydrofurane (3.71 g, 52.9 mmol) was added. The reaction mixture was stirred under nitrogen at 95 °C for 4 h. The mixture was cooled to RT, filtered and concentrated under vacuum and the residue taken-up with water (200 mL) and extracted with EtOAc (5 x 150 mL). The organic phase was dried ($Na_2SO_4$) and the crude product purified by flash chromatography eluting with PE / acetone (8 / 2) to afford **3** as yellow solid (3.54 g; 39 % over the two steps). $^1$H NMR (600 MHz, $CDCl_3$) δ: 8.30 (s, 1H, NH), 7.23 (dd, J = 9.10, 2.22 Hz, 1H, $ArH_6$), 7.16 (dd, J = 8.64, 2.27 Hz, 2H, $ArH_4$, $ArH_2$), 3.88 (t, J = 6.38 Hz, 2H, *CH₂*CH₂OH), 2.95 (t, J = 6.35 Hz, 1H, CH₂*CH₂*OH), 1.79 (s, 1H, OH). $^{13}$C NMR (151 MHz, $CDCl_3$) δ: 157.32 (d, $J_{C-F}$ = 239 Hz), 132.06, 128.25, 124.90, 114.26, 113.54, 104.36, 103.60, 62.61, 28.86. ESI-MS *m/z* $[M-H]^-$ 256, 258.

**Methyl 2-(8-bromo-1-ethyl-6-fluoro-1,3,4,9-tetrahydropyrano[3,4-b]indol-1-yl)acetate (5).**

To a solution of alcohol **3** (1.00 g, 2.7 mmol) in dry $CH_2Cl_2$ (25 mL) kept under nitrogen, methyl 3-oxopentanoate (**4**) (0.51 mL, 4.05 mmol) was added and the mixture was stirred at 0 °C for 10 min. Then, $BF_3 \cdot OEt_2$ (0.41 mL, 3.24 mmol) was added dropwise via a gas-tight syringe and the mixture stirred at RT for 5 h. The mixture was treated with $NaHCO_3$ saturated solution (25 mL) and extracted with $CH_2Cl_2$ (3 x 25 mL). The combined organic phases were washed with brine (40 mL), dried ($Na_2SO_4$) and evaporated under reduced pressure. The crude product was purified by flash chromatography using PE/EtOAc 95:5 as the eluent to obtain **5** as white solid (0.60 g; 60 %). $^1$H NMR (600 MHz, $CDCl_3$) δ: 9.29 (s, 1H, NH), 7.13 –7.09 (m, 2H, two signals: $ArH_5$, $ArH_7$), 4.07–4.02 and 3.94–3.75 (m, 2H, $H_{4a}$, $H_{4b}$), 3.75 (s, 3H, $COOCH_3$), 3.04 – 2.90 (m, 2H, $CH_2$COOMe), 2.80–2.76 and 2.71–2.67 (m, 2H, $H_{3a}$, $H_{3b}$), 2.17-2.11 and 2.04–1.98 (m, 2H, C*H₂*CH₃), 0.84 (t, J = 7.37 Hz, 3H, CH₂C*H₃*). $^{13}$C NMR (151 MHz, $CDCl_3$) δ: 173.11, 157.31 (d, $J_{C-F}$ = 238 Hz), 139.22, 131.48, 127.19 (d, $J_{C-F}$ = 10.1 Hz), 112.77 (d, $J_{C-F}$ = 29.1 Hz), 109.65 (d, $J_{C-F}$ = 4.8 Hz), 104.24 (d, $J_{C-F}$ = 12.3 Hz), 102.89 (d, $J_{C-F}$ = 23.2 Hz), 74.69, 60.52, 52.33, 42.69, 30.57, 22.48, 7.63. ESI-MS *m/z* $[M-H]^-$ 370, 372.

**Methyl 2-(1-ethyl-6-fluoro-8-(4,4,5,5-tetramethyl-1,3,2-dioxaborolan-2-yl)-1,3,4,9-tetrahydropyrano[3,4-b]indol-1-yl)acetate (6).**

In a two neck round-bottom flask, the bromoester **5** (0.60 g, 1.62 mmol), bis(pinacolato)diboron (0.82 g, 3.24 mmol) and potassium acetate (0.64 g, 6.48 mmol) were dissolved in 1,4-dioxane and stirred for 10 min at RT. To the obtained mixture, $Pd_2(dba)_3$ (22.3 mg, 0.024 mmol) and $PCy_3$ (31.8 mg, 0.011 mmol) were added and the mixture was stirred for other 10 minutes at RT. The mixture was heated at 100 °C under nitrogen overnight. The mixture was filtered under reduced pressure on a celite pad and concentrated under reduced pressure to give the crude product. The product was purified by flash chromatography (eluent PE/acetone 98:2) to give **6** as a white solid (0.55 g; 81 % yield). $^1$H NMR (600 MHz, $CDCl_3$) δ: 9.77 (s, 1H, NH), 7.29 (dd, J = 9.45, 2.57 Hz, 1H, $ArH_7$), 7.23

(dd, J = 9.43, 2.58 Hz, 1H, $ArH_5$), 4.07-4.01 and 3.95-3.90 (m, 2H, $H_{3a}$, $H_{3b}$), 3.71 (s, 3H, $COOC\mathit{H_3}$), 3.00 – 2.89 (m, 2H, $C\mathit{H_{2a}}COOMe$, $C\mathit{H_{2b}}COOMe$), 2.83-2.76 and 2.73-2.68 (m, 2H, $H_{4a}$, $H_{4b}$), 2.18 – 2.06 (m, 2H, $C\mathit{H_2}CH_3$), 1.41 (d, J = 8.11 Hz, 12H, $CH_3$ pinacol), 0.85 (t, J = 7.37 Hz, 3H, $CH_2C\mathit{H_3}$). $^{13}C$ NMR (151 MHz, $CDCl_3$) δ: 172.37, 157.60 (d, $J_{C-F}$ = 234 Hz), 138.36, 137.62, 126.21 (d, $J_{C-F}$ = 9.0 Hz, C8), 115.72 (d, $J_{C-F}$ = 24.3 Hz), 107.47 (d, $J_{C-F}$ = 4.64 Hz), 106.96 (d, $J_{C-F}$ = 23.8 Hz), 84.20, 83.64, 74.88, 60.59, 51.77, 42.51, 30.64, 25.16, 25.03, 22.35, 7.74. ESI-MS *m/z* $[M-H]^-$ 416.

**Methyl 2-(8-benzyl-1-ethyl-6-fluoro-1,3,4,9-tetrahydropyrano[3,4-b]indol-1-yl)acetate (8).**

In a screw-capped sealed tube, to a solution of **6** (0.300 g, 0.72 mmol) in THF and water (v/v 1/1) (7 mL), $Pd(dppf)Cl_2$ (25.2 mg, 0.036 mmol), $Na_2CO_3$ (190 mg, 1.80 mmol) and (bromomethyl)benzene (0.085 mL, 0.72 mmol) were added. The reaction was heated at 40 °C overnight. The mixture was then filtered on a büchner funnel and the filtrate was extracted with $CH_2Cl_2$ (3 x 20 mL). The combined organic layers were dried over $Na_2SO_4$ and evaporated to dryness. The crude product was purified by flash chromatography (eluent PE/EtOAc 9:1) to give **8** as a white solid (0.219 g; 80 % yield). $^1H$ NMR (600 MHz, $CDCl_3$) δ: 8.93 (s, 1H, NH), 7.32 – 7.19 (m, 5H, H2', 3', 4', 5', 6'), 7.02 (dd, J = 9.51, 2.60 Hz, 1H, $ArH_7$), 6.75 (dd, J = 9.51, 2.60 Hz $ArH_5$), 4.17 (s, 2H, $CH_2Ph$), 4.03 - 3.99 and 3.92-3.88 (m, 2H, $H_{3a}$, $H_{3b}$, 3.65 (s, 3H, $COOC\mathit{H_3}$), 2.96 – 2.82 (m, 2H, $C\mathit{H_2}COOMe$), 2.79-2.74 and 2.70-2.66 (m, 2H, $H_{4a}$, $H_{4b}$), 2.05-2.03 and 1.95-1.88 (m, 2H, $C\mathit{H_{2a}}CH_3$, $C\mathit{H_{2b}}CH_3$), 0.76 (t, J = 7.38 Hz, 3H, $CH_2C\mathit{H_3}$). $^{13}C$ NMR (151 MHz, $CDCl_3$) δ: 173.03, 157.96 (d, $J_{C-F}$ = 234 Hz), 139.15, 138.02, 131.22, 129.02, 128.78, 126.70 (d, $J_{C-F}$ = 9.9 Hz), 126.57, 124.99 (d, $J_{C-F}$ = 9.1 Hz), 110.66 (d, $J_{C-F}$ = 26.2 Hz), 108.61 (d, $J_{C-F}$ = 4.7 Hz), 101.56 (d, $J_{C-F}$ = 23.2 Hz), 74.63, 60.57, 52.09, 42.52, 38.00, 30.61, 22.40, 7.62. ESI-MS *m/z* $[M-H]^-$ 380.

**2-(8-benzyl-1-ethyl-6-fluoro-1,3,4,9-tetrahydropyrano[3,4-b]indol-1-yl)acetic acid (CXT29).**

To a solution of compound **8** (0.290 g, 0.76 mmol) in 1,4-dioxane (2.5 mL), an aqueous solution of LiOH 3M (2.5 ml, 7.6 mmol) was added and the reaction mixture was stirred at RT overnight. The reaction was quenched by addition of a solution of HCl 2M until acidic pH. The mixture was extracted with EtOAc (3 x 20 mL). The extracts were dried over $Na_2SO_4$ and evaporated to dryness. The crude product was purified by reverse-phase chromatography (Biotage SP, column AQ C18 spherical 20-35 μm 100 A 20 g) using $CH_3CN$/ $H_2O$ + 0.1 % TFA 6:4 as the eluent to give CXT29 as a white solid (0.128 g; 46 % yield). $^1H$ NMR (600 MHz, $CDCl_3$) δ: 8.29 (s, 1H, NH), 7.29 – 7.24 (m, 2H, $H_{2',6'}$), 7.23 – 7.17 (m, 3H, two signals: $H_{4'}$ and $H_{3',5'}$), 7.04 (dd, J = 9.15, 2.43 Hz, 1H, $ArH_7$), 6.76 (dd, J = 9.97, 2.41 Hz, 1H, $ArH_5$), 4.11 (s, 2H, $CH_2Ph$), 4.09– 4.05 and 4.01 – 3.97 (m, 2H, $H_{3a, H3b}$), 2.98 – 2.88 (m, 2H, $C\mathit{H_2}COOH$), 2.82 – 2.70 (m, 2H, $H_4$), 2.00 – 1.87 (m, 2H, $C\mathit{H_2}CH_3$), 0.76 (t, J = 7.35 Hz, 3H, $CH_2C\mathit{H_3}$). $^{13}C$ NMR (151 MHz, $CDCl_3$) δ: 175.40, 158.07 (d, $J_{C-F}$ = 235 Hz), 139.09,136.72, 131.42, 128.92, 128.87, 126.79 (d, $J_{C-F}$ = 10.07 Hz), 126.72, 124.93 (d, $J_{C-F}$ = 8.82 Hz), 111.15 (d, $J_{C-F}$ = 26.2 Hz), 108.76 (d, $J_{C-F}$ = 4.56 Hz), 101.76 (d, $J_{C-F}$ = 23.4 Hz), 75.08, 60.83, 42.49, 38.00, 30.88, 22.20, 7.69. ESI-MS *m/z* $[M-H]^-$ 366.

**Stability in simulated gastrointestinal fluids (SGF, SIF)**

The simulated gastrointestinal fluids were prepared according to USP specifications [Test solutions, United States Pharmacopoeia 28/National Formulary 23, 2005, USP-NF Online accessed 14 February, 2005]. A solution of compound (10 mM in DMSO) was added to simulated gastric fluid (SGF-without pepsin: 2.0 g/L sodium chloride and 2.917 g/L HCl, pH 1.2) or SIF (SIF-without pancreatine: 2.3 g/L of disodium hydrogen orthophosphate, 0.19 g/L of potassium dihydrogen

orthophosphate and 8.0 g/L of sodium chloride in sufficient water to produce 1000 mL, adjusted to pH 6.8) preheated to 37 °C. The resulting 100 µM solutions were maintained at 37 ± 0.5 °C; at appropriate time intervals, a 20 µL aliquot was withdrawn and analyzed by RP-HPLC (see below). Stability in SGF and SIF was evaluated as the percentage of unchanged compound during 24 h of incubation (results expressed as % of unmodified compound at 4, 6, 24 h).

**Stability in human serum**

A solution of compound (10 mM in DMSO) was added to human serum (sterile-filtered from human male AB plasma, USA origin, sterile-filtered, Sigma–Aldrich) preheated to 37 °C. Resulting solution (100 µM) was incubated at 37 ± 0.5 °C and at appropriate time intervals, 300 µL of reaction mixture was withdrawn and added to 300 µL of $CH_3CN$ containing 0.1% TFA in order to deproteinize the serum. Sample was sonicated, vortexed and then centrifuged for 10 minutes at 2150 *g*. The clear supernatant was filtered by 0.45 µm PTFE filters (Alltech) and analyzed by RP- HPLC (see below). Stability in human serum was evaluated as the percentage of unchanged compound during 24 h of incubation (results expressed as % of unmodified compound at 4, 6, 24 h).

**HPLC analysis**

The reverse-phase HPLC procedure allowed separation and quantitation of CXT29 and any degradation products. HPLC analysis was performed with a HP 1100 chromatograph system (Agilent Technologies, Palo Alto, CA, USA) equipped with a quaternary pump (model G1311A), a membrane degasser (G1379A), a diode-array detector (DAD) (model G1315B) integrated in the HP1100 system. Data analysis was processed using a HP ChemStation system (Agilent Technologies). The analytical column was a ZORBAX SB-Phenyl (250 × 4.6 mm, 5 µm; Agilent). The mobile phase consisted of $CH_3CN$ 0.1% TFA and 0.1% aqueous TFA, 70/30 w/w%, at flow rate = 1.0 mL/min. The injection volume was 20 µL (Rheodyne, Cotati, CA). The column effluent was monitored at 280 nm referenced against a 700 nm wavelength. Quantitation of compound was done using calibration curve obtained by analyzing standard solutions of compound; the linearity of the calibration curve was determined in a concentration range of 1-100 µM ($r^2 > 0.99$)

**Table S1. Stability of CXT29 in SGF, SIF and human serum.** Stability of **CXT29** in simulated gastric fluid (SGF), in simulated intestinal fluid (SIF), and in human serum during 24 hours incubation at 37°C. Results are reported as % of the unmodified compound at 4, 6 and 24 hours; data are means ± SD of three independent experiments. CXT29 is considered stable when its measured concentration remained within 95–105% of the initial value, with an RSD not exceeding 2%

| **CXT29 incubation time** | **% of compound ± RSD** | | |
|---|---|---|---|
| | **SGF, pH 1.2** | **SIF, pH 6.8** | **human serum** |
| **4 hours** | 99 ± 1 | 100 ± 1 | 100 ± 1 |
| **6 hours** | 98 ± 1 | 99 ± 1 | 100 ± 1 |
| **24 hours** | 97 ± 2 | 99 ± 1 | 99 ± 1 |

**Figure S1. Stability of CXT29 in SGF**. RP-HPLC chromatograms of CXT29 incubated in simulated gastric fluid (SGF, pH 1.2) at 37 °C: (a) immediately after sample preparation (0 h) and (b) after 24 h of incubation. One representative experiment out of three independent experiments is shown.

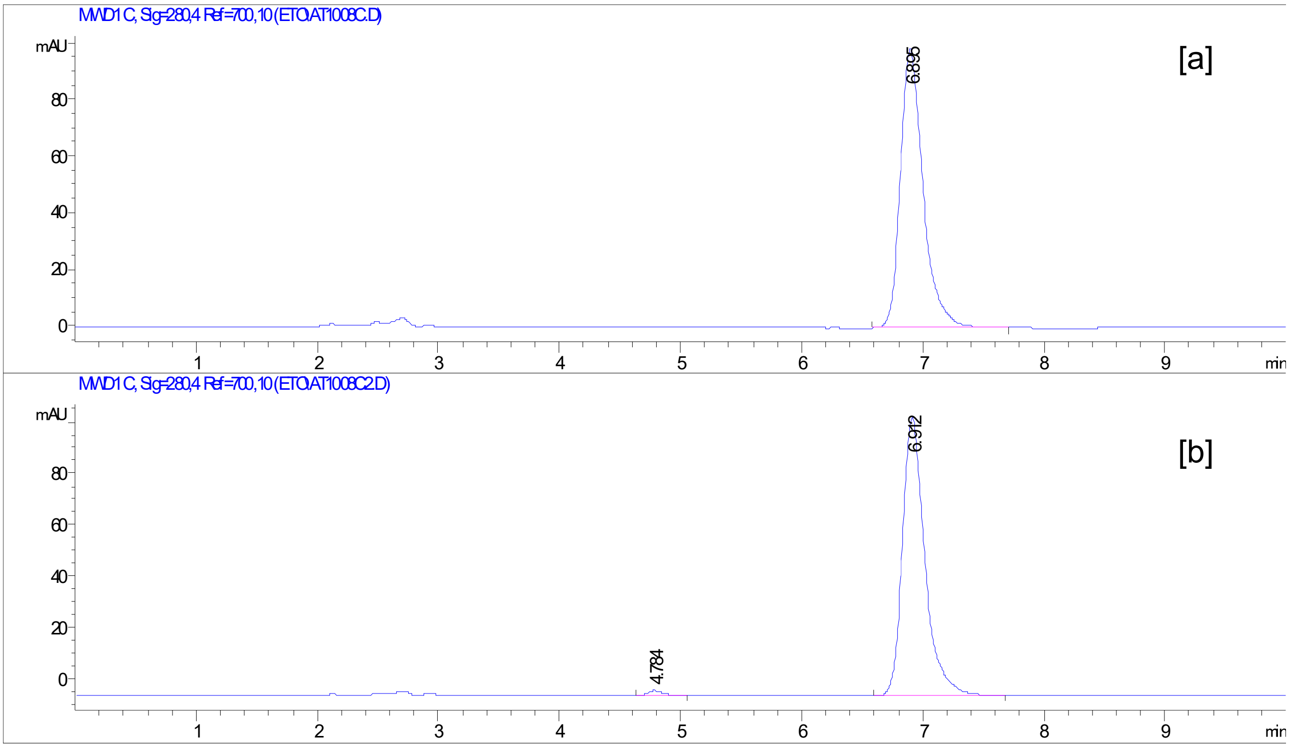

**Figure S2. Stability of CXT29 in SIF.** RP-HPLC chromatograms of CXT29 incubated in simulated intestinal fluid (SIF, pH 6.8) at 37 °C: (a) immediately after sample preparation (0 h) and (b) after 24 h of incubation. One representative experiment out of three independent experiments is shown.

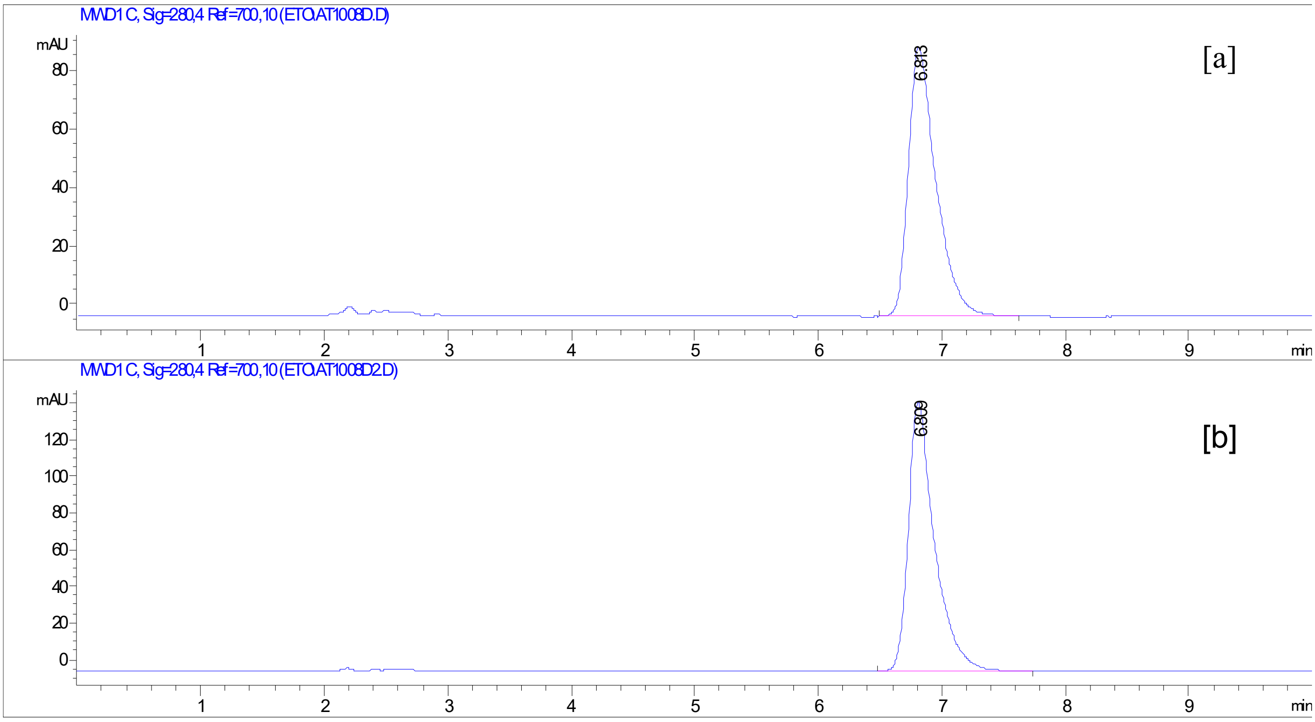


**Figure S3. Stability of CXT29 in human serum.** RP-HPLC chromatograms of CXT29 incubated in human serum at 37 °C: (a) immediately after sample preparation (0 h) and (b) after 24 h of incubation. One representative experiment out of three independent experiments is shown.

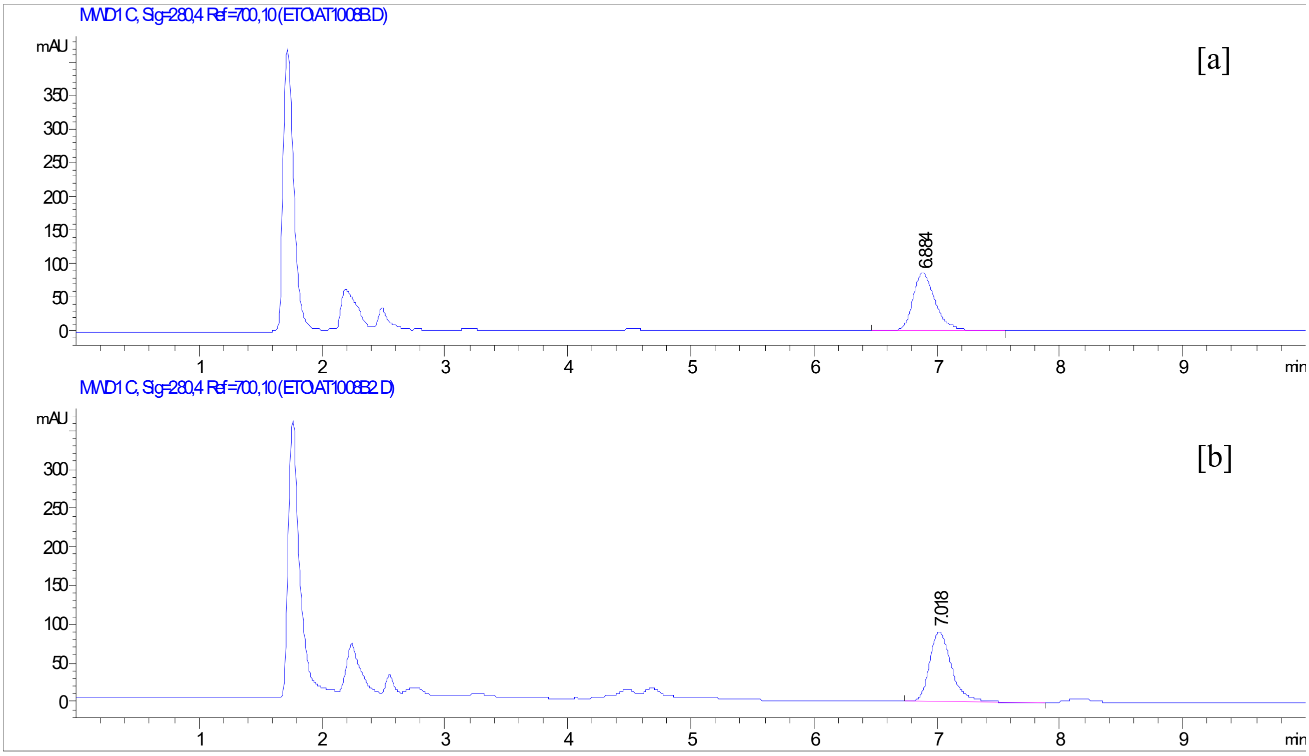

Supplement: Supplementary file 1 [file molecules-31-03403-s001.zip › molecules-4460567-supplementary.pdf]
